# Bioinformatics-based identification of glycolysis-related signatures associated with drug resistance and prognosis in lung adenocarcinoma

**DOI:** 10.3389/fonc.2026.1871582

**Published:** 2026-07-15

**Authors:** Qian Zheng, Yunxiao Liu, Tian Li, Xinge Zhang, Yadi Geng, Zhaolin Chen, Jing Zhou, Wenhao Fan, Yong Wang, Lei Zhang, Yuzhu Cao

**Affiliations:** 1 Department of Pharmacy, Anhui Institute of Medicine, Hefei, Anhui, China; 2 Department of Pharmacy, The First Affliated Hospital of University of Science and Technology of China, Division of Life Sciences and Medicine, University of Science and Technology of China, Hefei, China; 3 Anhui Provincial Key Laboratory of Precision Pharmaceutical Preparations and Clinical Pharmacy, The First Affliated Hospital of University of Science and Technology of China, Hefei, China; 4 Department of Medical Oncology, The First Affiliated Hospital of University of Science and Technology of China, Division of Life Sciences and Medicine, University of Science and Technology of China, Hefei, Anhui, China; 5 Department of Pharmacy, Fuyang Hospital of Anhui Medical University, Fuyang, Anhui, China

**Keywords:** glycolysis, lung adenocarcinoma, precise treatment, prognosis, therapeutic resistance

## Abstract

**Background:**

Drug resistance and poor clinical outcomes in lung adenocarcinoma (LUAD) necessitate robust biomarkers for personalized therapy. Glycolysis reprogramming is a hallmark of cancer, but its clinical utility remains incompletely defined.

**Methods:**

We integrated TCGA and GEO transcriptomic data with Weighted gene co−expression network analysis (WGCNA), least absolute shrinkage and selection operator (LASSO), and multivariate Cox regression to construct a glycolysis−related prognostic signature. A nomogram combining the risk score with clinicopathological factors was developed. Drug sensitivity was predicted using the pRRophetic algorithm. qRT−PCR and xenograft models using A549 and cisplatin−resistant A549/DDP cells validated the expression of candidate genes.

**Results:**

Patients stratified by glycolysis-related risk scores exhibited significantly distinct survival outcomes, and the glycolysis-based signature functioned as an independent prognostic factor for overall survival in LUAD. The nomogram demonstrated robust predictive performance and effectively estimated patient sensitivity to three commonly used conventional chemotherapeutic agents. *In vitro* and *in vivo* studies using A549 cells and their cisplatin-resistant derivative A549/DDP revealed aberrant expression of *VIPR1*, *ADRB2*, *RXFP1*, *PDGFB*, *WNT3A*, and *SPRY1* in the resistant cell line and in corresponding xenograft tumor tissues. These findings suggest that glycolytic activity is closely associated with both drug resistance and clinical prognosis in LUAD.

**Conclusions:**

This study identifies a glycolysis-related gene signature with demonstrable utility for prognostic stratification and therapeutic response prediction in LUAD. The proposed integrative model holds promise for enhancing precision treatment decision-making through optimized risk assessment and rational selection of chemotherapeutic regimens.

## Introduction

LUAD, the predominant histological subtype of non–small cell lung cancer (NSCLC), exhibits a persistently rising global incidence and remains a leading cause of cancer-related mortality (1). Despite significant advances in diagnostic and therapeutic modalities, long-term survival for LUAD patients remains unsatisfactory, with reported 5-year survival rates ranging from approximately 4% to 17% (2).

Conventional chemotherapy regimens for LUAD are largely non-targeted and frequently associated with substantial systemic toxicity, necessitating repeated treatment cycles and resulting in pronounced deterioration in patients’ quality of life. Moreover, therapeutic efficacy is highly variable, with only a subset of patients deriving meaningful clinical benefit (3). Although the emergence of molecularly targeted therapies and immunotherapies has expanded treatment options for LUAD, overall response rates remain unsatisfactory, and treatment resistance and adverse effects continue to limit durable clinical outcomes (4, 5).

The limited effectiveness of current therapeutic approaches is largely attributable to the pronounced heterogeneity of LUAD. Significant interpatient differences exist in molecular characteristics, tumor progression dynamics, and treatment sensitivity, posing major challenges to standardized treatment strategies (6, 7). Consequently, the identification of robust molecular biomarkers capable of stratifying patients by prognosis and therapeutic response has become a central focus in LUAD research, with the goal of enabling individualized treatment and improving clinical outcomes (8).

In this context, bioinformatics-driven analyses of large-scale genomic data have emerged as powerful tools for uncovering prognostically relevant molecular signatures. In the present study, we applied comprehensive bioinformatics and statistical approaches to systematically characterize the molecular landscape of LUAD and to develop a predictive model for patient prognosis and treatment response. Through integrative analysis of gene expression profiles, we identified key biomarkers significantly associated with clinical outcomes, with particular emphasis on glycolysis-related molecular features, which were further demonstrated to serve as independent prognostic indicators.

Glycolysis constitutes a canonical hallmark of cancer metabolism and plays a pivotal role in tumor initiation, progression, and adaptation to microenvironmental stress (9). Most solid tumors, including LUAD, undergo a metabolic shift toward aerobic glycolysis—notably the Warburg effect—to satisfy the heightened energetic and biosynthetic demands imposed by rapid proliferation and a heterogeneous tumor microenvironment (TME) (10). Even under hypoxic conditions, glycolysis remains the predominant pathway for ATP generation in tumor cells (11). Beyond energy production, glycolytic intermediates serve as precursors for macromolecular biosynthesis, support tumor invasion and metastasis, and contribute to the development of drug resistance (12). Accumulating evidence suggests that dysregulated glycolysis is closely associated with tumor aggressiveness and therapeutic resistance, highlighting its potential value as both a prognostic biomarker and a therapeutic target.

Based on these considerations, this study sought to define glycolysis-related gene signatures in LUAD and to evaluate their prognostic and predictive relevance. By integrating molecular signatures with clinicopathological variables, we constructed a comprehensive predictive model designed to enhance risk stratification accuracy and support individualized therapeutic decision-making for patients with LUAD.

## Materials and methods

### Data acquisition/dataset preparation and data processing

Transcriptomic and clinical data for LUAD were obtained from the TCGA and GEO databases. Samples were retained if they met the following criteria: (1) complete information on survival time, survival status, gender, age, and tumor stage; and (2) survival time ≥ 90 days. A total of 456 TCGA−LUAD samples and two GEO datasets (GSE72094, n = 369; GSE14814, n = 133) were included in the final analysis. TCGA−LUAD RNA−seq data (FPKM−normalized) and corresponding clinical information were downloaded from the UCSC Xena browser (https://xena.ucsc.edu). GEO datasets were accessed via the NCBI GEO repository. All expression data were log_2_−transformed and quantile−normalized. Batch effects between TCGA and GEO cohorts were corrected using the ComBat algorithm implemented in the sva R package.

### Candidate selection and signature establishment

Based on clinical data, TCGA-LUAD transcriptome data, and hallmark gene sets (cancer signature gene sets) data from the MsigDB database (http://www.gsea-msigdb.org/gsea/msigdb/). Univariate Cox regression analysis was performed to identify pathways associated with overall survival in LUAD. Penalized regression using LASSO with ten−fold cross−validation (cv.glmnet) was then applied. The optimal penalty parameter was selected at λ_min = 0.01215, corresponding to the minimum partial likelihood deviance.

WGCNA was performed to identify gene modules co−expressed with the glycolysis phenotype. A total of 19 prognostic genes were identified based on the association between gene modules and clinical outcomes.

The Kaplan-Meier survival analysis was used to evaluate the prognostic prediction of the model when patients in the training and validation sets were classified into high- and low-risk groups. Risk assessment model construction was performed using 19 prognostic genes: $Risk\;score=\sum_{i=1}^{N}Exp_{i}\times W_{i}$.The prognostic risk values were calculated for each patient according to the formula, and the patients were classified into high and low-risk groups. Kaplan-Meier survival analysis was used to evaluate the prognostic prediction of the model when patients in the training and validation sets were classified into high and low-risk groups.

### Bioinformatics and statistical analyses

Gene set variation analysis (GSVA) was performed using hallmark gene sets (h.all.v7.4.symbols.gmt) downloaded from the Molecular Signatures Database (MsigDB; http://www.gsea-msigdb.org/gsea/msigdb/). GSVA enrichment scores were calculated for each pathway and combined with clinical survival data. Univariate Cox and LASSO regression analyses were then applied to identify pathways significantly associated with overall survival (P<0.05).

Gene module analysis of TCGA lung cancer transcriptomic data was performed using WGCNA analysis, and the result was that glycolysis in GSVA was correlated with gene modules, and the pink module with the highest correlation with glycolysis was obtained (correlation of -0.54, P<0.05). The pink module contained a total of 314 genes. Combining TCGA lung cancer transcriptome data, clinical overall survival time, and survival status data, COX univariate and Lasso regression analyses were performed to obtain 19 prognostic genes involved in glycolysis in lung adenocarcinoma (P<0.05).

A risk assessment model was constructed using the formula: $Risk\;score=\sum_{i=1}^{N}Exp_{i}\times W_{i}$, where Exp was the expression of each mRNA, and W was the regression coefficient (coef) of each mRNA in Lasso regression. The prognostic risk value of each patient was calculated according to the formula, and then the sum of the risk values ​​was calculated. The median is the cutoff value to classify patients into high and low-risk groups. Kaplan-Meier survival analysis was used to evaluate the prognostic prediction effect of the model when the patients in the training and validation sets were divided into high-risk and low-risk groups. The timeROC package was used to evaluate the performance of this model at different time endpoints (3, 5 years). To predict performance, use the RMS package for nomogram drawing, and use the bootstrap internal validation method to verify the accuracy of the nomogram model. Normalization and batch correction were used to eliminate systematic bias when integrating or comparing TCGA and GEO expression data.

DESeq2 was used to analyze transcriptome differences between high and low-risk groups, and the screening criteria were padj<0.05 and |log_2_FC|>1; GISTIC 2.0 software was used to analyze copy number variation data of TCGA lung adenocarcinoma; R package ChAMP was used to analyze copy number variation. TCGA lung adenocarcinoma methylation data were used for methylation analysis, and the screening criteria were P Value < 10–15 and |log_2_FC|<0.1; the R package pRRophetic was used to evaluate 4 common targeted drugs in lung cancer treatment (cisplatin, chemosensitivity to gefitinib, gemcitabine, and doxorubicin).

### A549、A549/DDP xenograft mouse model establishment

Four−week−old male BALB/c nude mice were randomly divided into two groups (n = 6 per group; the experiment was repeated twice). A549 cells (4×10^6^ cells in 0.2 mL PBS) and A549/DDP cells (4×10^6^ cells in 0.2 mL PBS) were inoculated subcutaneously into the left inguinal region. Tumor volume was measured every 2 days using digital calipers and calculated as: volume = ½ × (length × width²). After four weeks, mice were euthanized, and tumor tissues were collected for histological examination and RNA extraction. No chemotherapeutic agents were administered during the observation period, as this experiment aimed to characterize baseline molecular differences between parental and acquired resistant cell lines. All animal experiments were performed in accordance with the ARRIVE guidelines and approved by the Institutional Animal Care and Use Committee (approval no. [2025-N(A)-0167]).

### RNA isolation and qRT-PCR analysis

Total RNA was extracted from cells and tissues using TRIzol reagent (Invitrogen, CA, USA). RNA was reverse−transcribed into cDNA using HiScript^®^ III RT SuperMix (Invitrogen, CA, USA). qRT−PCR was performed using the SYBR Green PCR kit (Vazyme, Nanjing, China) on a real−PCR system. GAPDH was used as the internal control. Each experiment was performed in triplicate. Quantitative data are expressed as mean ± standard deviation (SD). Statistical comparisons were analyzed using one−way analysis of variance (ANOVA) followed by Dunnett’s test. P < 0.05 was considered statistically significant. The primer sequences used are as follows:

*GAPDH* (179 bp): (sense) 5’-AGCAAGAGCACAAGAGGAAG-3’ and (antisense) 5’- GGTTGAGCACAGGGTACTTT -3’;

*VIPR1* (153 bp): (sense) 5’- TGGATGAACTCTGTGTGGTGC -3’ and (antisense) 5’- AGTTTCCTCATCTCTGCCGTC -3’;

*ADRB2* (169 bp): (sense) 5’- GGGTCTTTCAGGAGGCCAAA -3’ and (antisense) 5’- ATGCCTAACGTCTTGAGGGC -3’;

*RXFP1* (128 bp): (sense) 5’- TGCATGGATGTGACTAACCCA -3’ and (antisense) 5’- CGTTACAGTGCAGGAGCTGA -3’;

*PDGFB* (120 bp): (sense) 5’- GCGCCCATTTTTCATTCCCT -3’ and (antisense) 5’- CCGGTTTTCTCTTTGCAGCG -3’;

*WNT3A* (350 bp): (sense) 5’-TCCGCTTCTGCAGGAACTAC-3’ and (antisense) 5’- GAACTCCCGAGACACCATCC -3’;

*SPRY1* (283 bp): (sense) 5’-ATTTGCAATCTTTGCATTAGGCAT-3’and (antisense) 5’- GGCATGCATCTGAAATCCCTT -3’.

## Results

### Overview of the study design

First, pathway-level survival analyses identified the glycolysis pathway as a significant predictor of overall survival in patients with LUAD. To further elucidate glycolysis-associated molecular features and construct a prognostic model, a systematic analytical framework integrating WGCNA, univariate Cox regression, and LASSO regression was applied.

The resulting glycolysis-related risk signature was subsequently evaluated alongside conventional clinicopathological prognostic factors using multivariate Cox regression analysis. To facilitate individualized risk estimation and visualization of prognostic outcomes, a nomogram incorporating both molecular risk scores and clinical variables was constructed. Finally, drug sensitivity analyses were performed using the R package pRRophetic to assess differences in predicted responses to four commonly used conventional chemotherapeutic drugs across distinct risk subgroups.

### Glycolysis is a major risk factor for overall survival in LUAD

Transcriptomic profiles and corresponding clinical data of LUAD patients were obtained from the TCGA-LUAD cohort and integrated with curated cancer-related gene sets downloaded from the Molecular Signatures Database (MSigDB). Pathway-level survival analysis was performed to evaluate the association between biological pathways and overall survival in LUAD.

**Figure 1:**
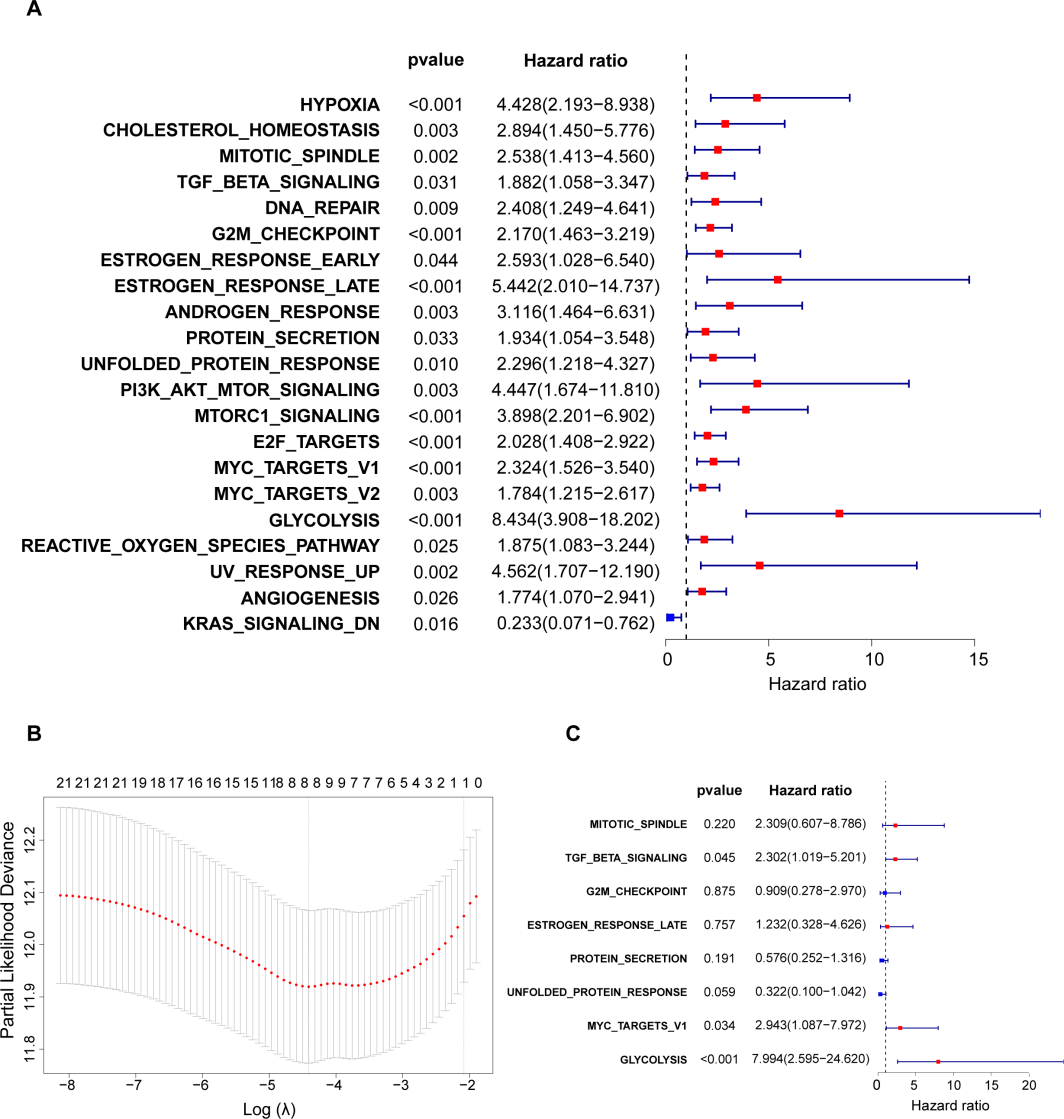
Glycolysis is a major risk factor for overall survival in patients with lung adenocarcinoma. **(A)** Univariate Cox regression analysis, with the pathway name in the first column, the p-value results in the second column, and the HR value in the third column, with values in parentheses denoting 95% confidence interval results. Risk factors are HR>1, and inhibitors are HR<1. Red dots and vertical lines reflect the log-partial likelihood function value + standard error corresponding to each in **(B)** The LASSO regression screening. The Log(λ) one standard error from the minimum is shown as the rightmost dashed line; **(C)** Cox multivariate findings, with values in parentheses denoting results with 95% confidence intervals. The first column of the results lists the pathway name, the second lists the p-value result, and the third lists the HR. HR<1: inhibitory factors, HR>1: risk factors.

Univariate Cox regression analysis identified 22 pathways significantly associated with overall survival (P < 0.05; **Figure 1A**). To reduce model overfitting and identify the most informative pathways, LASSO regression with ten-fold cross-validation was applied. When the optimal penalty parameter was selected (λ_min = 0.01215), eight survival-related pathways were retained based on minimized partial likelihood deviance (**Figure 1B**).

Subsequent multivariate Cox regression analysis demonstrated that the glycolysis pathway remained significantly associated with overall survival among the selected pathways, indicating that glycolysis represents an independent risk factor for prognosis in patients with LUAD (**Figure 1C**).

### Identification of glycolysis-related prognostic genes in LUAD

**Figure 2:**
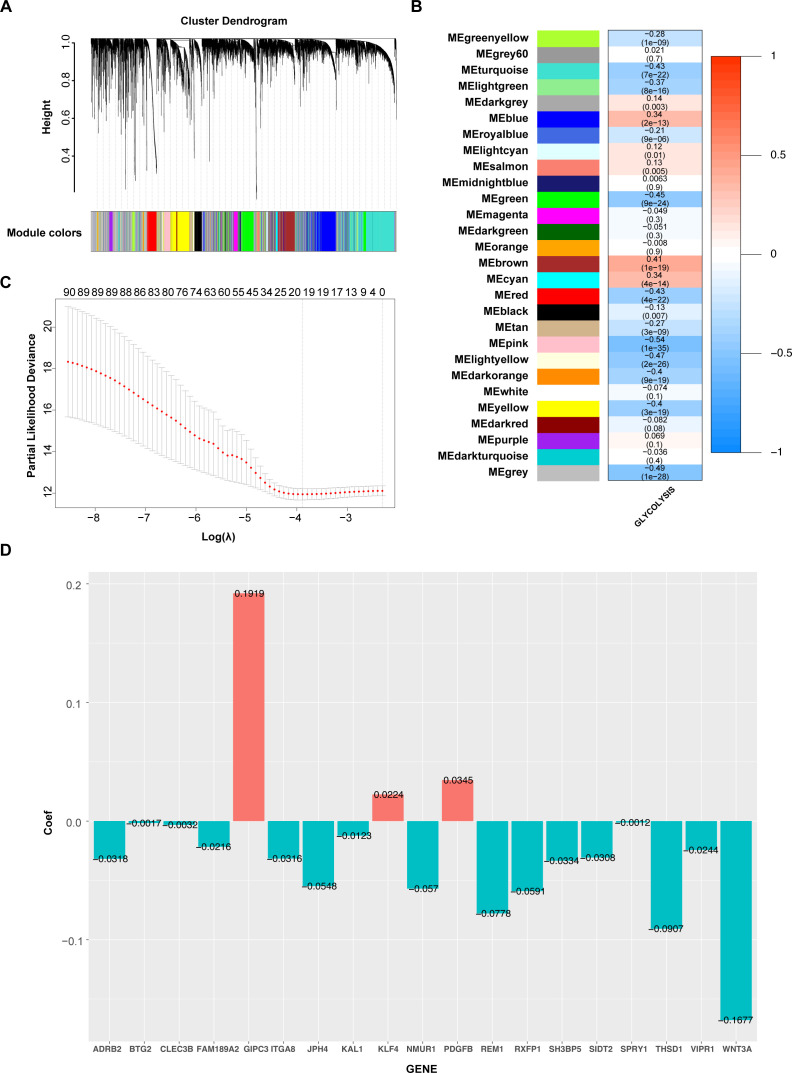
Prognostic genes involved in glycolysis in lung adenocarcinoma. The WGCNA hierarchical clustering in **(A)** displays each module; **(B)** Correlation analysis between the GLYCOLYSIS pathway and modules, with each cell including the correlation coefficient and the outcomes of a statistical test (numbers in brackets) between the two. Statistics were judged significant at P < 0.05. Vertical lines and red dots represent of Lasso regression screening genes; **(C)** show the value of the log partial likelihood function plus standard error for each λ. The Log(λ) one standard error from the minimum is shown by the dashed line on the right; **(D) **Regression coefficients for 19 prognostic genes.

To identify genes associated with glycolysis-related prognosis in LUAD, WGCNA was performed to construct gene co-expression networks based on transcriptomic data. A total of 28 gene modules were identified, with module sizes ranging from 47 to 2,493 genes (**Figure 2A**).

Module–trait relationship analysis revealed that the pink module exhibited the strongest correlation with glycolysis-related phenotypes (**Figure 2B**). This module comprised 314 genes and was therefore selected for subsequent analyses.

Within the pink module, univariate Cox regression analysis followed by LASSO regression identified 19 genes significantly associated with overall survival in LUAD. These genes were retained as candidate glycolysis-related prognostic markers and formed the basis for subsequent risk model construction (**Figures 2C, D**).

### Construction and validation of the glycolysis-related prognostic risk model

A prognostic risk assessment model was constructed based on the 19 glycolysis-related prognostic genes identified by LASSO regression: $Risk\;score=\sum_{i=1}^{N}Exp_{i}\times W_{i}$. The risk score for each patient was calculated as a weighted linear combination of gene expression levels, where Exp represents the normalized expression value of each gene and W denotes the corresponding LASSO regression coefficient. The LASSO regression coefficients (Coef values) for these 19 genes have been provided in **Supplementary Table 1**.

**Figure 3:**
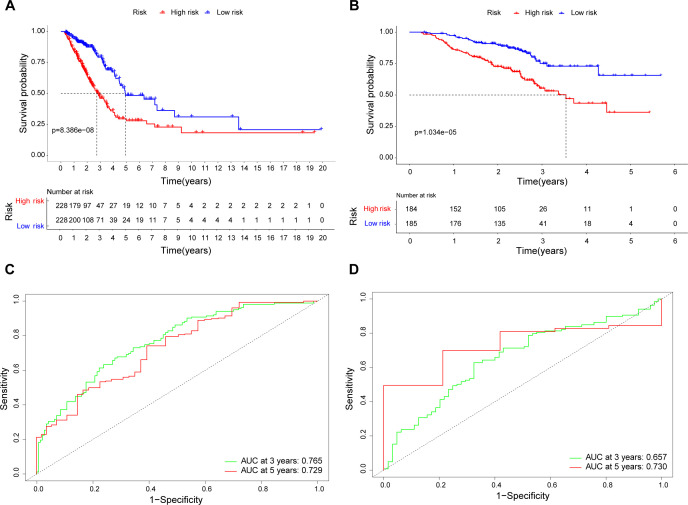
Model construction and validation of glycolysis-related prognostic genes. **(A)** The TCGA-LUAD survival curves for various risk categories; **(B)**Survival curves of different risk groups in GSE72094; **(C)**ROC curves were used to assess lung cancer survival rates at three and five years in the TCGA; **(D)**ROC curves were used to assess lung cancer survival rates at 3- and 5-year intervals in GSE72094. AUC values range from 0 to 1, with higher values indicating better predictive performance.

Using the median risk score as the cutoff, patients in both the training (TCGA-LUAD) and validation (GSE72094) cohorts were stratified into high-risk and low-risk groups. Kaplan–Meier survival analysis demonstrated that patients in the high-risk group exhibited significantly poorer overall survival compared with those in the low-risk group in both cohorts (P < 0.05; **Figures 3A, B**).

The predictive performance of the risk model was further evaluated using time-dependent receiver operating characteristic (ROC) analysis. In the TCGA-LUAD training cohort, the area under the curve (AUC) values for 3-year and 5-year overall survival were 0.765 and 0.729, respectively. Consistent results were observed in the validation cohort GSE72094, with AUC values of 0.657 at 3 years and 0.730 at 5 years (**Figures 3C, D**). These findings indicate that the 19-gene glycolysis-related signature exhibits stable and reliable prognostic performance across independent datasets.

We further performed validation using the independent external cohort GSE14814 from the GEO database, which included 133 eligible samples with complete survival follow-up information. Kaplan-Meier survival analysis showed that patients in the high-risk group had significantly worse overall survival (OS) than those in the low-risk group (log-rank P = 0.04244) (**Supplementary Figure 1A**). The model demonstrated favorable long-term survival predictive performance, with 3-year and 5-year AUC values of 0.608 and 0.613, respectively (**Supplementary Figure 1B**). A total of 13 of the 19 prognostic genes were detected in the external cohort. The risk scores calculated based on these genes showed a prognostic trend consistent with that observed in the training cohort, confirming the favorable robustness of the 19-gene signature model in independent samples.

### Comparison of prognostic and predictive value between the glycolysis-related risk score and conventional clinical factors

To determine whether the glycolysis-related risk score provided independent prognostic information beyond traditional clinical variables, multivariate Cox regression analyses were performed incorporating age, sex, tumor stage, and risk score.

**Figure 4:**
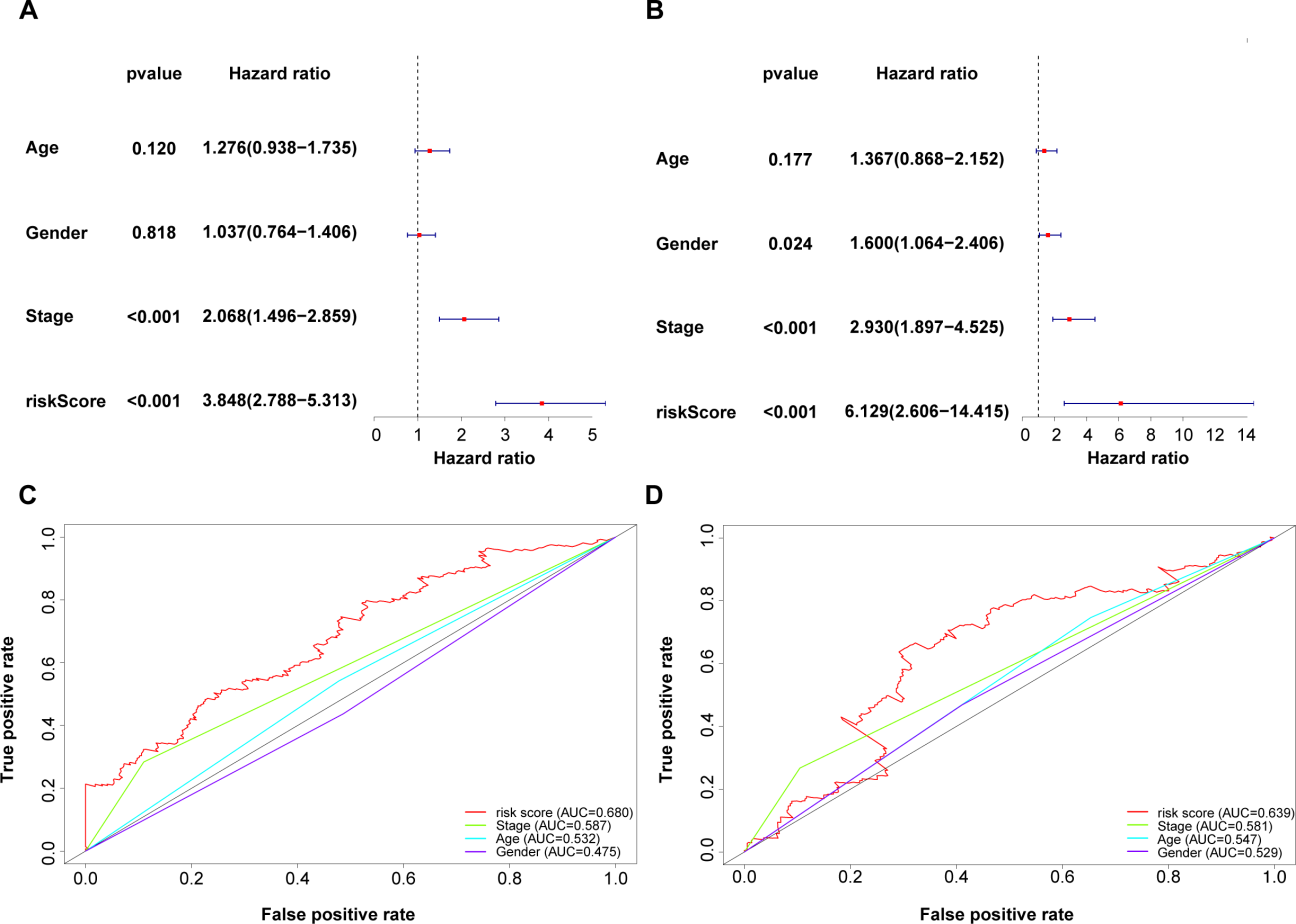
Comparison of the prognostic and predictive power of glycolysis-related prognostic risk scores with traditional features. **(A)** The cox multivariate results of Age, Gender, Stage, and riskScore in the training set of the TCGA, where the first column is the prognostic indicator, the second column is the result of the P value, and the third column is the HR value, in parenthesis. The figures show outcomes with a 95% confidence level. The risk factor is HR>1, the inhibiting factor is HR<1; **(B)** The cox multivariate results of Age, Gender, Stage, and riskScore in the validation set GSE72094, where the first column is the prognostic indicator, the second column is the result of the P value, and the third column is the HR value, in parenthesis. The figures show outcomes with a 95% confidence level. The risk factor is HR>1, the inhibiting factor is HR<1; **(C)** Training set TCGA multi-index ROC curve. **(D)** Validation set GSE72094 multi-index ROC curve.

In the TCGA-LUAD cohort, tumor stage (hazard ratio [HR] = 2.068) and risk score (HR = 3.848) were both significantly associated with overall survival. Similar results were observed in the GSE72094 validation cohort, where tumor stage remained significantly associated with survival (HR = 2.068). When analyses were conducted across LUAD patients, tumor stage and risk score yielded HRs of 2.930 and 6.129, respectively, with both associations reaching statistical significance (P < 0.01; **Figures 4A, B**). These results indicate that the glycolysis-related risk score functions as an independent prognostic factor for overall survival.

The discriminative ability of age, sex, tumor stage, and risk score for predicting 5-year overall survival was assessed using ROC curve analysis. In the TCGA-LUAD cohort, the AUC values for tumor stage and risk score were 0.587 and 0.680, respectively. In the validation cohort, the AUC values were 0.581 for tumor stage and 0.639 for risk score (**Figures 4C, D**). Among all evaluated variables, the risk score consistently demonstrated the highest AUC, indicating superior prognostic discrimination compared with conventional clinical factors.

### Evaluation of the nomogram incorporating glycolysis-related risk score and clinical variables

**Figure 5:**
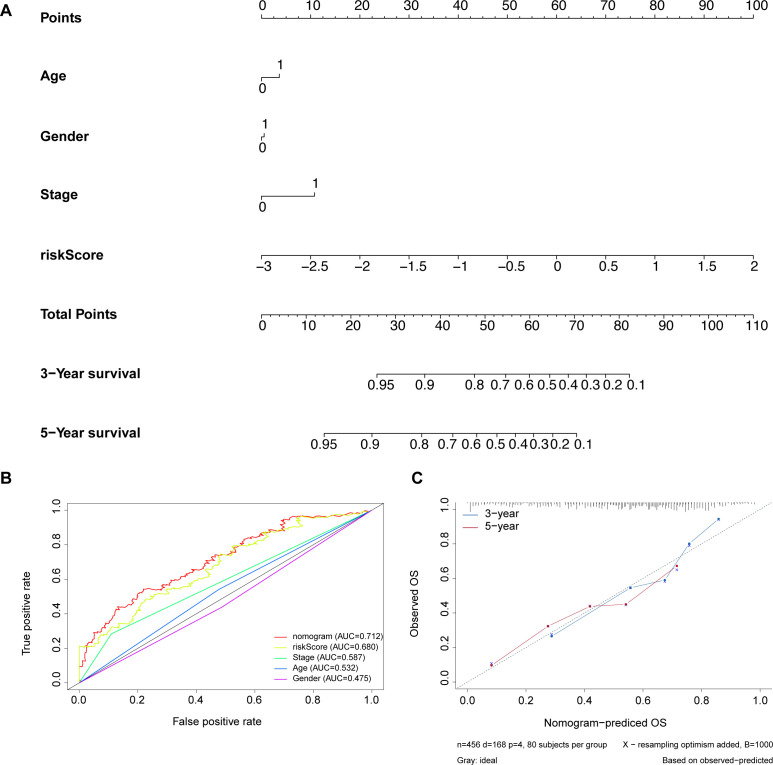
Nomogram drawing and evaluation. **(A)** A prognostic nomogram model that projects overall survival at 3 and 5 years based on risk levels and other clinical data; **(B)** Nomogram and multi-index ROC curve; **(C)** Calibration curves for estimating the total survival time across three and five years.

To facilitate individualized survival prediction, a nomogram was constructed by integrating the glycolysis-related risk score with relevant clinicopathological variables based on multivariate Cox regression analysis. The nomogram indicated that the risk score contributed the greatest prognostic weight, followed by tumor stage (**Figure 5A**).

The predictive accuracy of the nomogram was assessed using ROC analysis. The nomogram achieved an AUC of 0.712, outperforming models based on tumor stage alone (AUC = 0.587) or risk score alone (AUC = 0.680), indicating improved prognostic discrimination (**Figure 5B**).

Internal validation of the nomogram was performed using bootstrap resampling. Calibration curve analysis demonstrated good concordance between predicted and observed survival probabilities, confirming the robustness and reliability of the nomogram for survival prediction in LUAD patients (**Figure 5C**).

### Multi-omics characterization of high- and low-risk LUAD subgroups

**Figure 6:**
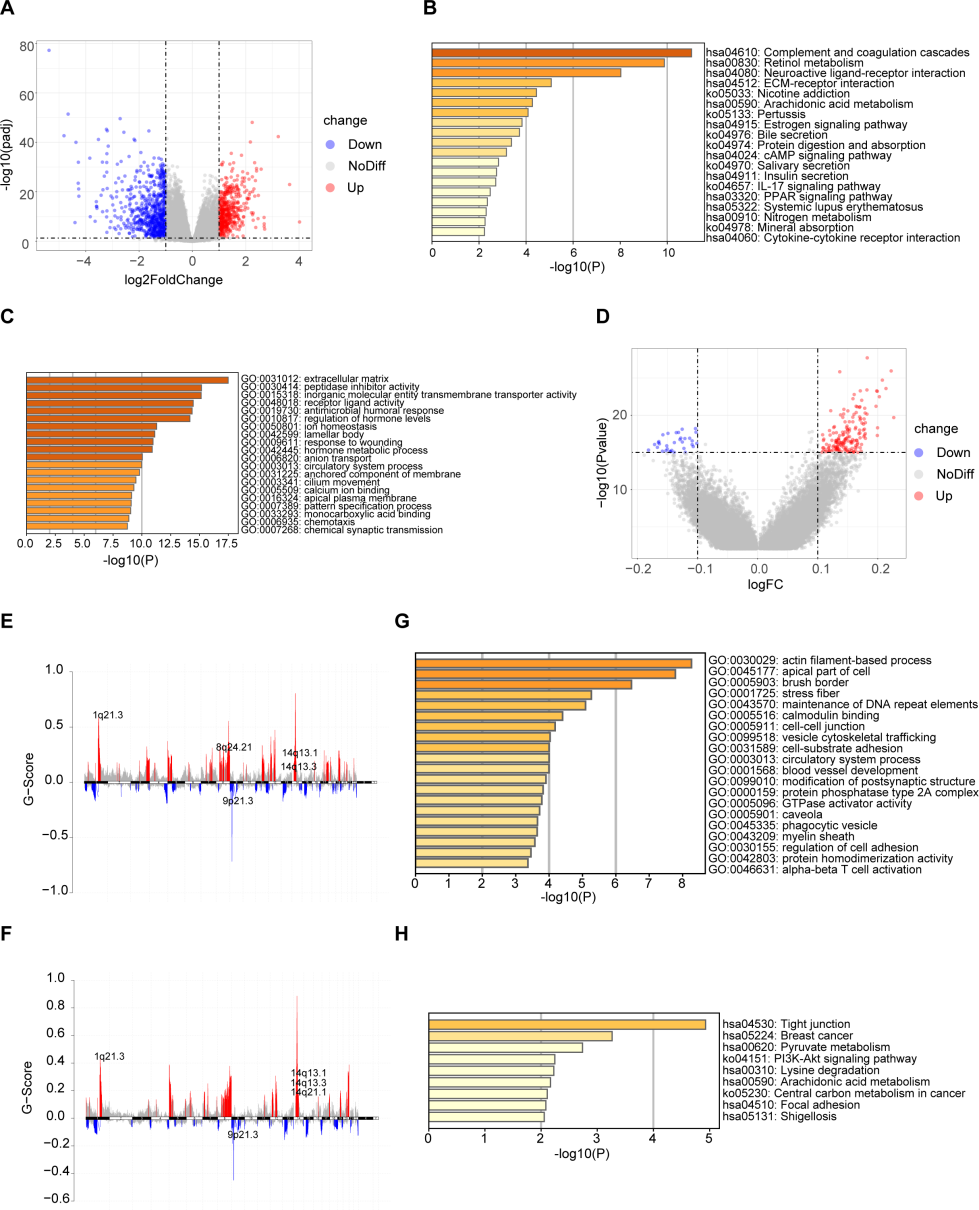
Multi−omics characterization of high− and low−risk subgroups. **(A)** Volcano plot of differentially expressed genes (DEGs) between risk groups; red = upregulated, blue = downregulated (padj < 0.05, |log_2_FC| > 1); **(B)** GO enrichment analysis of DEGs; **(C)** KEGG pathway enrichment analysis of DEGs; **(D)** Methylation differential analysis; red = hypermethylated, blue = hypomethylated in high−risk group; **(E)** Copy number variation (CNV) analysis in high−risk subgroup; **(F)** CNV analysis in low−risk subgroup. **(D–F)** represent genome−wide analyses and do not specifically interrogate the 19−gene signature, as methylation and CNV data were not available for the same cohort. **(G)** Methylation GO enrichment analysis; **(H)** Methylation KEGG enrichment analysis.

Differential gene expression analysis between high-risk and low-risk subgroups was performed using DESeq2, with thresholds of adjusted P value (padj) < 0.05 and |log_2_ FC| > 1. A total of 1,203 differentially expressed genes (DEGs) were identified, of which 490 were upregulated and 713 were downregulated in the low-risk group compared with the high-risk group (**Figure 6A**).

Functional enrichment analysis of DEGs was conducted using Metascape. Gene Ontology (GO) analysis revealed significant enrichment in biological processes related to extracellular matrix organization, receptor–ligand interactions, hormone regulation and metabolism, ion transport, and ion binding (**Figure 6B**). Kyoto Encyclopedia of Genes and Genomes (KEGG) pathway analysis further indicated that these DEGs were primarily enriched in estrogen signaling, cyclic AMP (cAMP) signaling, IL-17 signaling, PPAR signaling, and other cancer-related pathways (**Figure 6C**).

Copy number variation (CNV) analysis was performed on TCGA-LUAD samples using GISTIC 2.0. Both copy number gains and deletions were observed in high- and low-risk subgroups; however, the high-risk subgroup exhibited a greater extent and frequency of CNV events compared with the low-risk subgroup (**Figures 6E, F**).

DNA methylation profiles from the TCGA-LUAD cohort were analyzed using the ChAMP R package, applying thresholds of P < 1 × 10⁻¹^5^ and |log_2_ FC| > 0.1. Compared with the low-risk group, 167 hypermethylated and 33 hypomethylated CpG sites were identified in the high-risk group (**Figure 6D**). These sites were annotated to 175 genes. Subsequent integration with differentially expressed genes identified a subset of differentially methylated and expressed genes. GO enrichment analysis of these genes demonstrated significant enrichment in pathways related to cell attachment, cell adhesion, cellular transport, and vascular development (**Figure 6G**). KEGG pathway analysis indicated predominant enrichment in metabolic pathways and the PI3K–Akt signaling pathway (**Figure 6H**).

### Glycolysis-related risk signature predicts therapeutic response

Drug sensitivity analyses were conducted using the R package pRRophetic to estimate the half-maximal inhibitory concentration (IC_50_) values of four commonly used chemotherapeutic or conventional chemotherapeutic agents in LUAD treatment, including cisplatin, gefitinib, gemcitabine, and doxorubicin.

**Figure 7:**
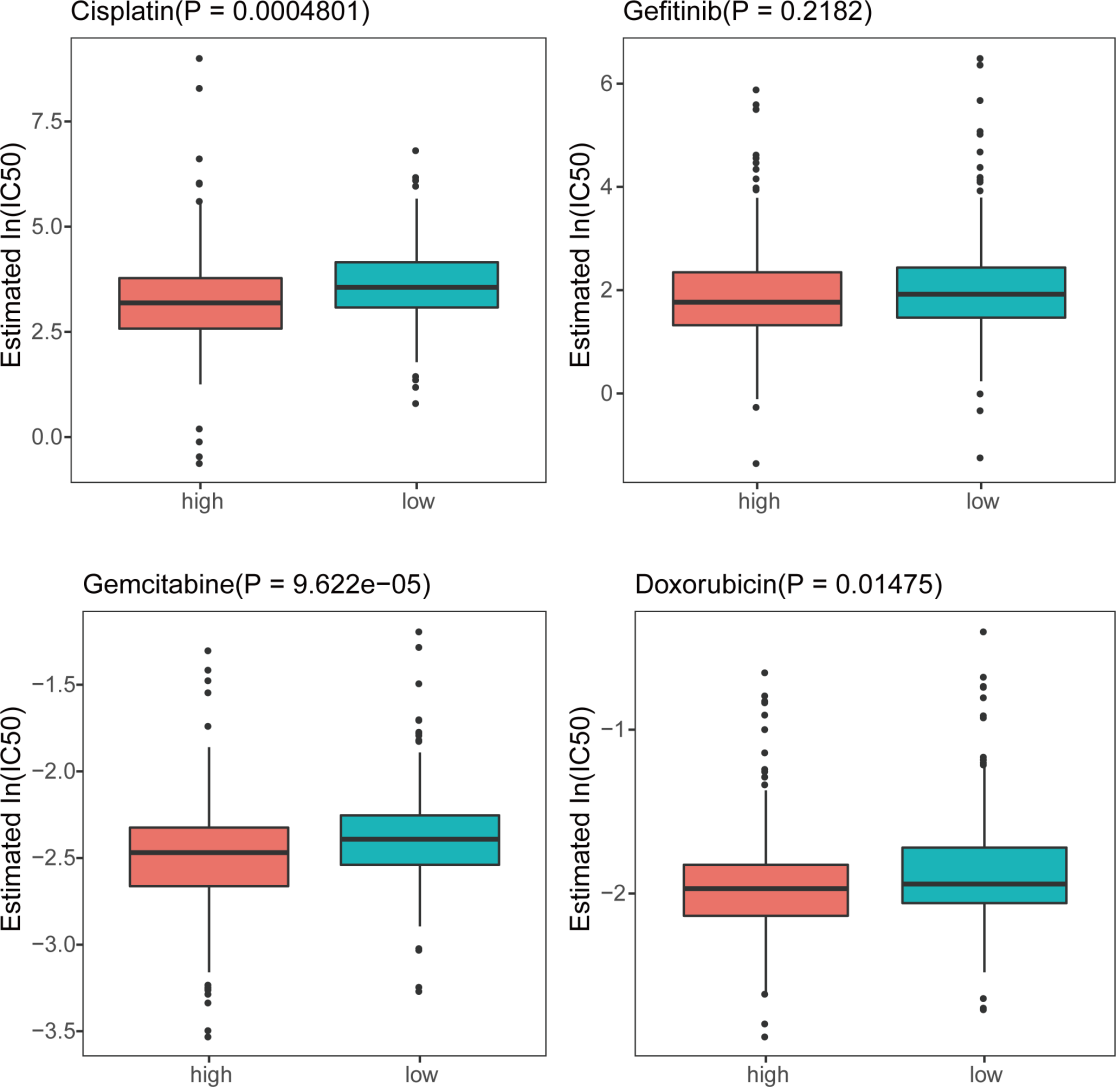
Predicted drug sensitivity between high‑ and low‑risk groups. Estimated IC_50_ values for cisplatin, gefitinib, gemcitabine, and doxorubicin were calculated using the pRRophetic algorithm. Lower IC_50_ in the high‑risk group indicates enhanced predicted baseline chemosensitivity. P<0.05, significant. These predictions are derived from treatment‑naïve transcriptomic profiles and do not represent acquired resistance.

Significant differences in predicted IC_50_ values were observed between high-risk and low-risk subgroups for cisplatin, gemcitabine, and doxorubicin, whereas no significant difference was detected for gefitinib. Notably, the high-risk subgroup exhibited lower IC_50_ values for these agents, indicating increased predicted sensitivity to commonly used chemotherapeutic drugs (**Figure 7**).

Drug sensitivity analysis predicted that patients in the high−risk group had lower IC_50_ values for cisplatin, gemcitabine, and doxorubicin, suggesting enhanced baseline chemosensitivity (**Figure 7**). This finding, while initially counterintuitive, aligns with the concept that rapidly proliferating, glycolytically active cancer cells are more vulnerable to cytotoxic agents that target dividing cells. However, this baseline sensitivity does not preclude the development of acquired resistance following prolonged drug exposure, which involves additional epigenetic and metabolic adaptations.

### *In vitro* and *In vivo* validation of glycolysis-related prognostic markers

**Figure 8:**
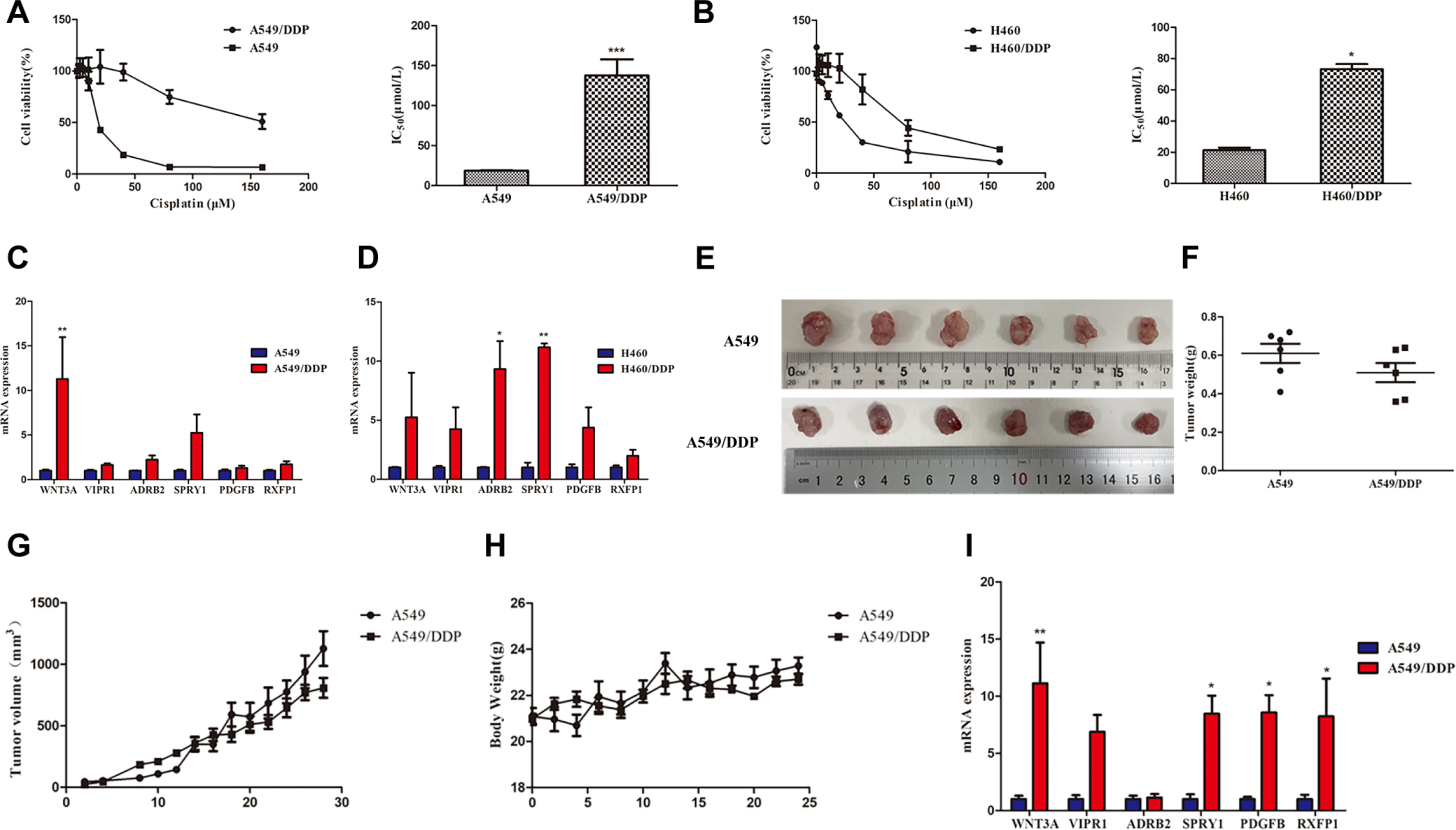
*In vitro* and *in vivo* validation of glycolysis−related candidate genes. **(A)** Different concentrations of cisplatin were used on A549, and A549/DDP cells and CCK-8 calculated IC_50_ after 24h to detect drug resistance; **(B)** H460 and H460/DDP cells were treated with different concentrations of cisplatin, and cck8 calculated IC_50_ after 24 h to detect drug resistance; **(C, D)** mRNA expression levels of *WNT3A*, *VIPR1*, *ADRB2*, *SPRY1*, *PDGFB* and *RXFP1* detected by qRT-PCR in A549/A549/DDP **(C)** and H460/H460/DDP **(D)** cells detected by qRT−PCR, *p<0.05, **p<0.01, ***p <0.001; **(E–G)** Tumor volume and tumor weight changes in mice; **(H)** Body weight changes in mice; **(I)** qRT−PCR analysis of *WNT3A*, *VIPR1*, *ADRB2*, *SPRY1*, *PDGFB* and *RXFP1* mRNA levelsin A549− and A549/DDP−derived tumor tissues. Data are shown as mean ± SD (n = 3 technical replicates; n = 6 biological replicates per group *in vivo*). *p<0.05, **p<0.01, ***p<0.001. Statistical comparisons were analyzed based on one‐way analysis of variance (ANOVA) followed by Dunnett’s test. A p‐value of < 0.05 indicates a significant difference.

To experimentally validate the association between glycolysis-related signatures and drug resistance, the inhibitory effects of cisplatin on cell proliferation were assessed in human lung cancer cell lines A549 and H460, as well as their cisplatin-resistant derivatives A549/DDP and H460/DDP. Dose–response analyses revealed IC_50_ values of 18.51 μM and 21.36 μM for A549 and H460 cells, respectively, compared with markedly higher IC_50_ values of 137.63 μM and 73.17 μM for A549/DDP and H460/DDP cells (**Figures 8A, B**). The *in vitro* experimental results presented here contradict the aforementioned conclusion that high-risk patients exhibit a lower IC_50_. This apparent paradox is mechanistically plausible: highly glycolytic, rapidly proliferating cells in *in vitro* monolayer systems exhibit heightened sensitivity to cell-cycle-targeting drugs (13, 14). However, in clinical *in vivo* settings, these hyper-glycolytic tumors create a severely hypoxic, highly acidotic, and immunosuppressive tumor microenvironment (TME), which hinders drug penetration and promotes stemness, ultimately resulting in systemic failure and resistance (15). Additionally, our *in vitro* and *in vivo* validation models utilize acquired cisplatin-resistant cell lines (A549/DDP). Once selected under chronic drug pressure, these cells undergo secondary epigenetic and metabolic reprogramming, surpassing their baseline hyper-proliferative drug sensitivity.

Through literature research, six genes (*WNT3A*, *VIPR1*, *ADRB2*, *SPRY1*, *PDGFB*, and *RXFP1*) related to lung cancer and the regulation of glycolytic metabolic pathways were identified among the 19 genes. Notably, six of the 19 prognostic genes are non-enzymatic regulators that modulate glycolytic flux via receptor signaling or transcriptional control (16–21).Quantitative real-time PCR (qRT-PCR) analysis demonstrated that the mRNA expression levels of *WNT3A*, *VIPR1*, *ADRB2*, *SPRY1*, *PDGFB*, and *RXFP1* were elevated in cisplatin-resistant cell lines compared with their parental counterparts. Among these, *WNT3A*, *ADRB2*, and *SPRY1* exhibited statistically significant upregulation (**Figures 8C, D**).

To further validate these findings *in vivo*, xenograft models were established using A549 and A549/DDP cells. Representative tumor images are shown in **Figure 8E**. Over a four-week observation period, no significant differences were observed in tumor volume, tumor weight, or body weight between the two groups (**Figures 8F–H**). Successful tumor establishment was confirmed by hematoxylin and eosin (H&E) staining and Ki-67 immunohistochemistry (**Supplementary Figure 2**). Because both A549 (parental) and A549/DDP (resistant) cells are highly malignant, they exhibit similarly high baseline growth capacities in the absence of chemotherapy select-pressure, which explains why no significant differences in vehicle-treated tumor volumes, weights, or mouse body weights were observed.

qRT-PCR analysis of tumor tissues demonstrated that the mRNA expression levels of *VIPR1*, *SPRY1*, *PDGFB*, and *RXFP1* were significantly higher in tumors derived from A549/DDP cells compared with those from A549 cells (**Figure 8I**), further supporting the association between glycolysis-related gene expression and cisplatin resistance in LUAD.

## Discussion

Metabolic reprogramming, particularly aerobic glycolysis, is a recognized hallmark of cancer. However, translating this knowledge into clinically actionable biomarkers remains challenging. Here, we provide a systematically derived glycolysis−related signature with dual utility in prognosis prediction and therapeutic response stratification.

It is well established that malignant cells exhibit a metabolic phenotype characterized by increased glycolysis, even under aerobic conditions (22). Although this phenomenon has been extensively documented, the biological and clinical implications of tumor dependence on glycolytic metabolism remain incompletely understood. Metabolic reprogramming is now recognized as a hallmark of cancer, enabling tumor cells to adapt to diverse microenvironmental stresses and sustain uncontrolled proliferation (23). Enhanced aerobic glycolysis supports rapid ATP production and provides metabolic intermediates required for biosynthetic processes, thereby conferring a selective growth advantage to cancer cells (24–26). Accumulating evidence has demonstrated a close association between glycolytic activity and tumor cell proliferation, survival, and disease progression across multiple cancer types (27).

In lung cancer, glycolysis has been implicated in promoting metastatic potential by facilitating cytoskeletal remodeling and enhancing cellular motility in both murine and human models (20). Persistent activation of glycolytic pathways has also been associated with malignant progression and poor clinical outcomes in NSCLC (28, 29). These observations suggest that dysregulated glycolysis contributes not only to tumor growth but also to treatment resistance, underscoring its potential relevance as a prognostic biomarker and therapeutic target (30). Accordingly, a deeper understanding of glycolysis-related molecular alterations may inform the development of metabolic-based therapeutic strategies, particularly when combined with existing anticancer treatments (31).

In the present study, we systematically constructed a glycolysis-related gene signature using integrated bioinformatics and statistical approaches and demonstrated that glycolysis-associated molecular features are strongly linked to prognosis in lung adenocarcinoma (LUAD). By incorporating transcriptomic profiling with survival analyses, we identified a risk score that effectively stratified patients into distinct prognostic subgroups. Importantly, the glycolysis-related risk score remained an independent predictor of overall survival after adjustment for conventional clinicopathological factors, highlighting its potential clinical utility beyond established prognostic indicators.

To further enhance individualized risk assessment, we developed a nomogram integrating the glycolysis-based risk score with clinical variables. This model exhibited improved predictive accuracy compared with traditional staging systems alone, suggesting that metabolic signatures may provide complementary prognostic information. In addition, drug sensitivity analyses indicated that the glycolysis-related risk signature was associated with differential responses to commonly used chemotherapeutic agents, supporting its potential role in guiding treatment selection and optimizing therapeutic strategies for LUAD patients. *In vitro* cell experiments have shown that the mRNA expression levels of *WNT3A*, *VIPR1*, *ADRB2*, *SPRY1*, *PDGFB*, and *RXFP1* are significantly higher in drug-resistant NSCLC cell lines compared to non-resistant cell lines, with particularly significant differences observed in *WNT3A*, *ADRB2*, and *SPRY1*. Xenograft mouse model experiments have demonstrated that, compared to tumor tissues from mice with non-resistant cell lines in the control group, the mRNA expression levels of *VIPR1*, *SPRY1*, *PDGFB*, and *RXFP1* are significantly elevated in tumors from drug-resistant NSCLC cell lines. This provides solid experimental support for the results of previous analyses.

Despite these strengths, several limitations should be acknowledged. First, this study was primarily based on retrospective analyses of publicly available datasets, which may introduce selection bias. Second, the dataset used in this study only provided transcriptomic (gene expression) data for lung cancer samples, and did not include DNA methylation or copy number variation (CNV) data for the same set of samples. Therefore, from a technical perspective, the current dataset does not permit direct analysis of methylation modifications or CNVs for the 19 core prognostic genes. This is a limitation of the present study. We believe that future studies should validate these findings in independent cohorts integrating multi-omics data, such as TCGA or GEO datasets and their matched methylation/CNV profiles. Finally, although *in vitro* and *in vivo* experiments supported the association between glycolysis-related genes and drug resistance, validation in large, well-characterized clinical cohorts is still required to confirm the translational applicability of the proposed model. Future studies incorporating prospective clinical samples and functional mechanistic investigations will be essential to further substantiate these findings (32).

## Conclusion

In conclusion, this glycolysis−related signature represents a promising tool for prognostic stratification and baseline chemosensitivity estimation in LUAD. Future studies should explore its integration with dynamic monitoring of metabolic changes during treatment to guide adaptive therapy.

## Data Availability

The original contributions presented in the study are included in the article/**Supplementary Material**. Further inquiries can be directed to the corresponding author.
