# Supplementary material for: Bioinformatics-based identification of glycolysis-related signatures associated with drug resistance and prognosis in lung adenocarcinoma
Source: Front Oncol. 2026 Jul 15;16:1871582. doi: 10.3389/fonc.2026.1871582 (PMC13414110; doi:10.3389/fonc.2026.1871582)

**Supplemental Table 1 Coef values of 19 genes**

| Gene | Coef |
| --- | --- |
| VIPR1 | -0.024374282369138 |
| KLF4 | 0.0223998398409141 |
| SH3BP5 | -0.0334197264878385 |
| THSD1 | -0.0907494897487668 |
| ADRB2 | -0.0317930717327305 |
| GIPC3 | 0.191934544373648 |
| KAL1 | -0.0122787261116708 |
| RXFP1 | -0.0590638143639546 |
| FAM189A2 | -0.0216151022772297 |
| REM1 | -0.0778202636018337 |
| SIDT2 | -0.0307675275953239 |
| PDGFB | 0.0344708925136771 |
| WNT3A | -0.167745164391787 |
| CLEC3B | -0.00321485174191162 |
| SPRY1 | -0.00122113251835855 |
| JPH4 | -0.0547871304215599 |
| ITGA8 | -0.0315739543605797 |
| NMUR1 | -0.056950483937011 |
| BTG2 | -0.00166156145428994 |

**Supplemental Figure 1 Supplementary validation of the glycolysis-related prognostic gene model construction**

(A) Survival curves of different risk groups in GSE14814**.** (B) ROC curves of the model were used to assess lung cancer survival rates at 3- and 5-year intervals in GSE14814. AUC is a crucial indication for assessing each of their markers' performance, and the closer it is to 1, the better the performance.

**
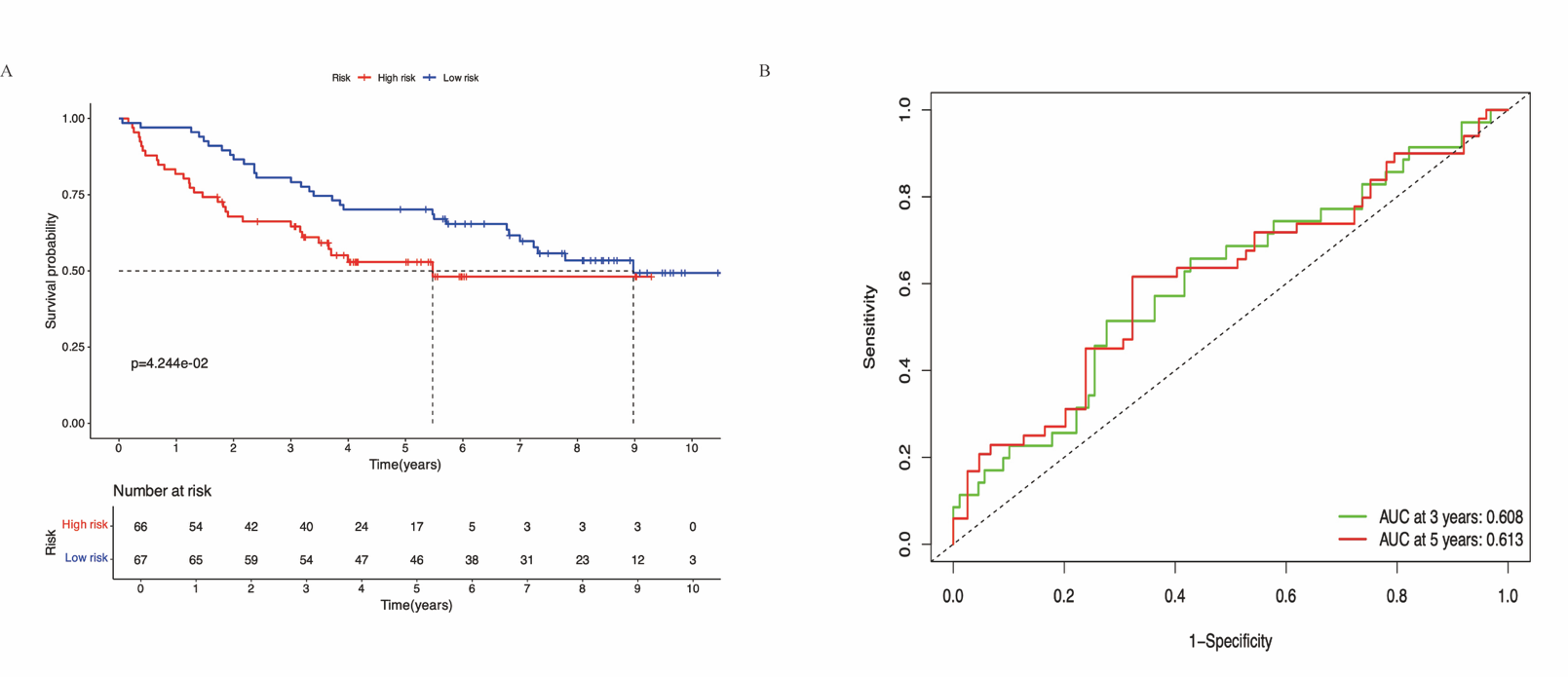
**

**Supplemental Figure 2 HE&Ki67 staining of mice**

(A) HE staining of A549 tumor-bearing mice (B) HE staining of A549/DDP tumor-bearing mice (C) Ki67 staining of A549 tumor-bearing mice (D) Ki67 staining of A549/DDP tumor-bearing mice


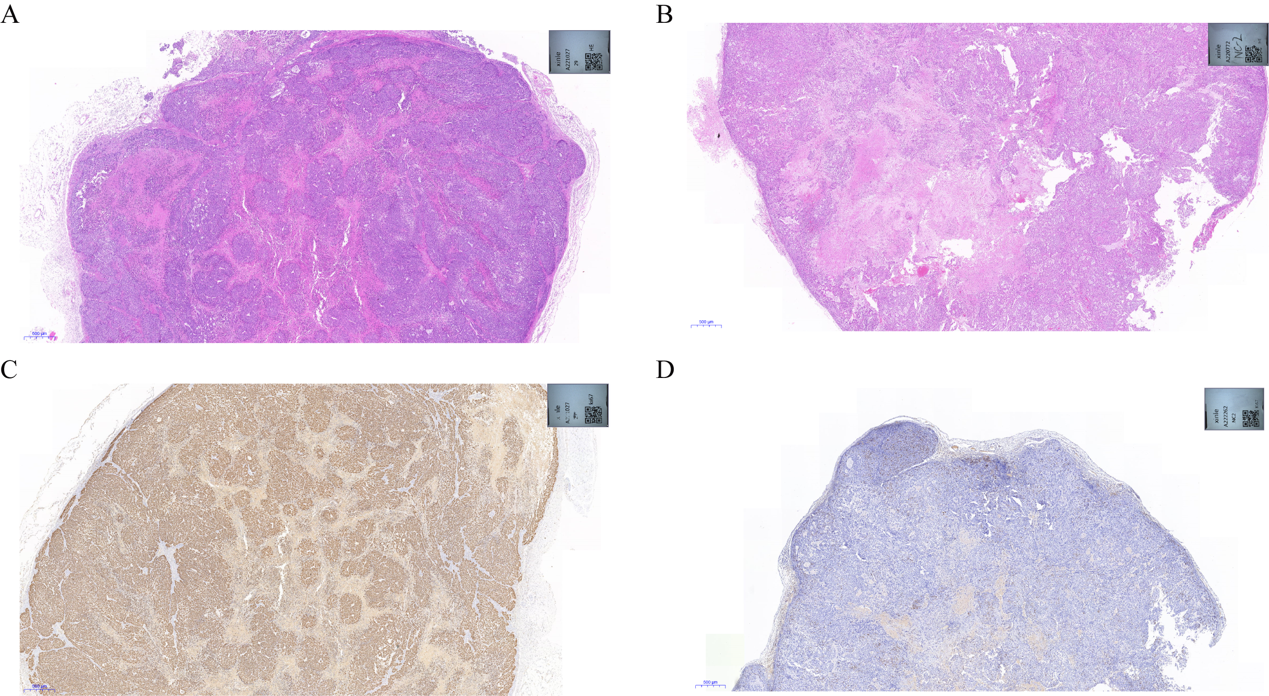

Supplement: Supplementary file 1 [file DataSheet1.docx]
